# Black–White Blood Pressure Disparities: Depressive Symptoms and Differential Vulnerability to Blood Lead

**DOI:** 10.1289/ehp.1104517

**Published:** 2012-10-25

**Authors:** Margaret T. Hicken, Gilbert C. Gee, Cathleen Connell, Rachel C. Snow, Jeffrey Morenoff, Howard Hu

**Affiliations:** 1 Department of Epidemiology, University of Michigan, Ann Arbor, Michigan, USA; 2 Department of Community Health Sciences, University of California, Los Angeles, Los Angeles, California, USA; 3 Department of Health Behavior and Health Education, and; 4 Department of Sociology, University of Michigan, Ann Arbor, Michigan, USA; 5 Dalla Lana School of Public Health, University of Toronto, Toronto, Ontario, Canada

**Keywords:** African Americans, depressive symptoms, health status disparities, hypertension, lead, psychosocial stress

## Abstract

Background: Blacks have higher hypertension rates than whites, but the reasons for these disparities are unknown. Differential vulnerability, through which stress alters vulnerability to the effects of environmental hazards, is an emergent notion in environmental health that may contribute to these disparities.

Objectives: We examined whether blacks and whites exhibit different associations between blood lead (BPb) and blood pressure (BP) and whether depressive symptoms may play a role.

Methods: Using the National Health and Nutrition Examination Survey 2005–2008, we regressed BP on the three-way interaction among race/ethnicity, BPb, and depressive symptoms in blacks and whites ≥ 20 years of age.

Results: Blacks but not whites showed a positive association between BPb and systolic blood pressure (SBP). The disparity in this association between blacks and whites appeared to be specific to the high depressive symptoms group. In the low depressive symptoms group, there was no significant black–white disparity (β_interaction_ = 0.9 mmHg; 95% CI: –0.9, 2.7). However, of those with high depressive symptoms, blacks and whites had 5.6 mmHg (95% CI: 2.0, 9.2) and 1.2 mmHg (95% CI: –0.5, 2.9) increases in SBP, respectively, in association with each doubling of BPb (β_interaction_ = 4.4 mmHg; 95% CI: 0.5, 8.3). The pattern of results was similar for diastolic blood pressure.

Conclusions: Our results suggest that depressive symptoms may contribute to the black–white disparity in the association between BPb and BP. Depressive symptoms may result, in part, from psychosocial stress. Our results support the notion that stress increases vulnerability to the health effects of environmental hazards and suggest that stress-related vulnerability may be an important determinant of racial/ethnic health disparities.

Black–white disparities in hypertension have been documented for decades (Heymsfield et al. 1977; Mensah et al. 2005). Recent nationwide prevalence estimates for hypertension in adults are at roughly 33% for whites but 43% for blacks (Lloyd-Jones et al. 2010). Although the disparities have been well characterized, the causes are poorly understood. Research suggests that unequal social factors and environmental exposures experienced by blacks and whites contribute substantially to racial/ethnic disparities in health, including hypertension (Cooper 1993; Lillie-Blanton and Laveist 1996; Morello-Frosch and Lopez 2006; Williams and Collins 2001).

There is an emergent notion in the environmental health literature that social factors and stress alter vulnerability to the harmful health effects of environmental exposures that then contribute to racial/ethnic health disparities (Gee and Payne-Sturges 2004). It may be that the greater levels of chronic social stressors, such as poverty, poor neighborhoods, and racial/ethnic discrimination, experienced by blacks compared to whites (Clark et al. 1999; Dressler 1996; Geronimus et al. 2007; Sternthal et al. 2011; Turner 2009; Williams et al. 2003), result in increased vulnerability to hypertensive effects of environmental hazards.

There is empirical support for the notion that social stressors and psychosocial stress moderate the association between environmental hazards, such as lead, and health. In other words, some studies have shown that the association between lead and health may differ by the level of stress. For example, a stronger inverse association between bone lead and cognitive outcomes has been reported for people who live in neighborhoods with high (compared to low) levels of psychosocial hazards, or who report high (compared to low) levels of perceived stress (Glass et al. 2009; Peters et al. 2010). In addition, the association between bone lead and blood pressure (BP) was stronger in men who reported high levels of perceived stress compared with men who reported low levels of perceived stress (Peters et al. 2007). Similarly, the association between blood lead (BPb) and hypertension was stronger in adults with high, compared to low, allostatic load scores, which are a measure of the cumulative dysfunction of physiological systems due to psychosocial stress (McEwen 1998; Zota et al. 2010).

Lead is causally related to a modest increase in BP and risk of hypertension in a dose-dependent manner (Ahamed and Siddiqui 2007; Cheng et al. 2001; Hu et al. 1996; Navas-Acien et al. 2007; Vaziri 2008). Since the legislation in the 1980s governing lead use, lead exposure has decreased substantially in the United States (Muntner et al. 2005; Pirkle et al. 1998). For example, unadjusted mean BPb levels for black and white women in the National Health and Nutrition Examination Survey (NHANES) II (1976–1980) were 13.2 μg/dL and 12.1 μg/dL, respectively (Sorel et al. 1991) compared with 2.3 μg/dL and 2.1 μg/dL in the NHANES III (1988–1994) (Den Hond et al. 2002). In addition, although BPb was associated with BP among both blacks and whites in NHANES II (Harlan 1988), the BPb–BP association was not present for white women in NHANES III, but remained large and statistically significant for black women (Den Hond et al. 2002; Muntner et al. 2005; Vupputuri et al. 2003), with a 1.2 mmHg increase in systolic BP estimated for every doubling of BPb (*p* < 0.01) (Den Hond et al. 2002). The black–white disparities in these patterns over time were similar for men. What might explain this new racial/ethnic disparity in the association between BPb and BP? In this study, we examined the role of stress in the stronger association between BPb and BP in black compared to whites. Specifically, our objectives were to *a*) document whether there continues to be a black–white disparity in the association between BPb and BP, and *b*) examine the mediating and moderating roles of depressive symptoms, as a proxy for stress, in this stronger association.

## Methods

*Data set*. We used data from two recent 2-year waves of the NHANES that include data collected from 2005 through 2008. The details of this survey are provided elsewhere [National Center for Health Statistics (NCHS) 2011]. Briefly, NHANES is currently an annual survey and clinical exam of a population-representative sample of roughly 5,000 noninstitutionalized U.S. residents ≥ 1 year of age administered by the Centers for Disease Control and Prevention. NHANES is approved by the National Center for Health Statistics Research ethics review board and all participants provide informed consent. For the present analysis, we included all adults ≥ 20 years of age who self-identified as either non-Hispanic black or non-Hispanic white. We excluded women who were pregnant because pregancy may affect BPb levels and BP. We also excluded those with missing information on any variables included in our models, yielding a final sample size of 4,470 (1,218 black and 3,252 white).

*Variables*. Systolic (SBP) and diastolic blood pressure (DBP) values were the average of up to three seated BP readings measured during the clinic examination. Whole BPb was measured using inductively coupled plasma mass spectrometry, and values below the limit of detection were imputed as the lower-bound of the detection limit (0.25 μg/dL) divided by the square root of 2 (i.e., 0.18 μg/dL) (NCHS 2008). We log-transformed BPb values because of heteroskedasticity and centered them before modeling interactions.

There are no measures of psychosocial stress in NHANES. Although depressive symptoms are not a direct measure of psychosocial stress, researchers argue that depressive symptoms may mark group differences in psychosocial stress and psychological health (Aneshensel et al. 1991; Mirowsky and Ross 2003). Depressive symptoms are measured in NHANES using the Patient Health Questionnaire (PHQ-9), a tool used for screening and monitoring depression as defined by the *Diagnostic and Statistical Manual of Mental Disorders*, *Fourth Edition* (DSM-IV) (Kroenke et al. 2001). The scale reliability coefficient, α, is 0.84. Because we found that the association between depressive symptoms and BP was nonlinear, we created a dichotomous variable based on the distribution of the scores in the total sample and coded it as: 0 = lower two tertiles (score < 3); 1 = highest tertile (score ≥ 3).

*Analytic approach*. To examine black–white differences in the association between BPb and BP, we regressed BP (SBP and DBP in separate models) on the interaction between race/ethnicity and log-transformed BPb. All models included lower-order terms for race/ethnicity (black vs. white) and BPb (log-transformed) plus a multiplicative interaction term between race/ethnicity and BPb. In addition, we adjusted for age, age^2^, and sex (model 1); everything in model 1 plus education (< high school, high school diploma, > high school) and family poverty income ratio (PIR; the ratio of family gross income to family census poverty threshold) (model 2); and everything in model 2 plus hematocrit, body mass index (BMI), heavy alcohol use [≥ 15 drinks/week and ≥ 5 drinks/day for men, ≥ 7 drinks/week and ≥ 4 drinks/day for women (National Institute on Alcohol Abuse and Alcoholism 2005)], smoking status (never, former, current), and self-report of a diagnosis of diabetes (model 3).

Although researchers generally report no black–white disparities in depressive symptoms or depression based on the PHQ-9 or other DSM-IV–related instruments (Gavin et al. 2010; Mezuk et al. 2010; Riolo et al. 2005), researchers have shown that, for a given level of depressive symptoms, blacks report more stress compared to whites (Keyes et al. 2011; Mezuk et al. 2010). Therefore, because the meaning of depressive symptoms with regard to stress may be different between the race/ethnicities, we examined the role of depressive symptoms and race/ethnicity in the association between BPb and BP by regressing BP on a three-way interaction among race/ethnicity, depressive symptoms, and BPb. Specifically, we modeled lower-order terms for race/ethnicity (black vs. white), BPb (log-transformed), depressive symptoms (high vs. low); pair-wise multiplicative interaction terms for race/ethnicity × BPb, race/ethnicity × depressive symptoms, and BPb × depressive symptoms; and a three-way interaction term for race/ethnicity × BPb × depressive symptoms.

We conducted numerous sensitivity analyses, beginning with an examination of different models of the association among race/ethnicity, depressive symptoms, and BPb. Specifically, we examined the potential for *a*) a mediated moderation and/or *b*) a moderated mediation among these variables (Muller et al. 2005). Beginning with an examination of mediated moderation, we examined the possibility that racial/ethnic disparities in the association between BPb and BP are mediated by (i.e., explained by or in the pathway of) racial/ethnic disparities in depressive symptoms. Briefly, if racial/ethnic disparities in depressive symptoms explain the racial/ethnic disparities in the association between BPb and BP, the regression term for the interaction between race/ethnicity and BPb should be reduced after the addition of the interaction between race/ethnicity and depressive symptoms to the model. The interaction terms for race/ethnicity and BPb did not change with the addition of the second interaction term, in either effect size or statistical significance (data not shown). We then continued with an examination of moderated mediation because some research has shown that lead is related to depression (Rhodes et al. 2003), and depression is also related to hypertension (Scalco et al. 2005). We examined the possibility that *a*) depressive symptoms mediate (i.e., explain or are in the pathway of) the association between BPb and BP, and *b*) this role of depressive symptoms is moderated (i.e., differs) by race/ethnicity. We did not observe a significant difference (*p* > 0.05) in the association between BPb and depressive symptoms according to race/ethnicity, which suggests that the association is not moderated by race/ethnicity (data not shown).

Next, because lead is stored in bone and poor bone health may result in the release of lead into the blood, we ran separate models adjusted for factors related to bone health and BP, specifically, menopausal status [in women only: premenopausal, menopausal, menopausal plus estradiol hormone replacement therapy (Kalkwarf et al. 2003)]; age (< 50 years, ≥ 50 years); and vitamin D intake (modeled as a continuous variable calculated from NHANES staff from dietary intake questionnaires). We also ran models adjusted for the intake of several nutritional factors that may be associated with BPb and BP, including both serum and dietary (in separate models) calcium, iron, potassium, and sodium (modeled as continuous variables calculated from laboratory values, for serum levels, and calculated values from dietary intake questionnaires, for dietary levels). Because 30% of blacks and 26% of whites in our sample report use of antihypertensive medication, we also ran models adjusted for antihypertensive medication use. In one model, we included a dummy variable for antihypertensive medication use. However, there is controversy over the inclusion of this variable in BP models because it is also a collider in the directed acyclic graph linking BP and numerous social factors such as education and poverty. Therefore, in another model, we added 10 mmHg to the SBP value of all those taking antihypertensive medication, as suggested in the literature (Tobin et al. 2005). The results from each of these models were similar to those of our more parsimonious models, so we do not report them.

All analyses were conducted in STATA version 11.0 (StataCorp, College Station, TX, USA. We employed NHANES sample weights to account for the complex survey design and aggregation of data over multiple years (NCHS 2006).

## Results

Mean SBP, but not mean DBP, was significantly higher in blacks than whites (Table 1). BPb levels for blacks and whites were similar (1.9 ± 2.2 μg/dL and 1.7 ± 0.9 μg/dL, respectively), but statistically different (*p* = 0.006). Thirty-nine percent of blacks are in the high depressive symptoms group, compared to 35% of whites. BPb levels according to low or high depressive symptoms were similar for both whites (1.7 ± 0.8 μg/dL in both groups, *p* = 0.755) and blacks (1.8 ± 2.1 μg/dL and 1.9 ± 1.8 μg/dL for the low and high depressive symptoms groups, respectively, *p* = 0.755) (data not shown).

**Table 1:** Sociodemographic and health characteristics by race/ethnicity, unadjusted, NHANES 2005–2008.

Characteristic	Black (n = 1,217)	White (n = 3,245)	p-Valuea
SBP (mmHg)	124.6 ± 20.6	121.7 ± 11.1	0.000
DBP (mmHg)	71.6 ± 15.8	70.8 ± 7.9	0.074
BPb (μg/dL)	1.9 ± 2.2	1.7 ± 0.9	0.006
Depressive symptoms score	3.2 ± 5.0	2.7 ± 2.4	0.014
High depressive symptomsb	475 (39)	1,136 (35)	0.092
Age (years)	42.2 ± 16.4	47.1 ± 10.8	0.000
Women	645 (53)	1,655 (51)	0.294
Education
< High school	280 (23)	357 (11)	0.000
High school	280 (23)	779 (24)	0.611
> High school	657 (54)	2,109 (65)	0.000
PIR	2.6 ± 1.7	3.5 ± 1.0	0.000
Diabetes	183 (15)	357 (11)	0.005
Smoking status
Never	730 (60)	1,590 (49)	0.000
Former	146 (12)	876 (27)	0.000
Current	341 (28)	779 (24)	0.068
Heavy alcohol use	158 (13)	584 (18)	0.010
BMI (kg/m2)	29.8 ± 8.2	28.1 ± 4.2	0.000
Hematocrit (%)	40.9 ± 5.2	42.5 ± 2.6	0.000
Values shown are mean ± SD or n (%). Results were weighted to account for complex survey design. ap-Values for black–white comparisons. bPHQ-9 score ≥ 3.						

In the overall sample, each doubling of BPb was associated with a 1.3 mmHg increase in SBP (95% CI: 0.1, 2.5) and a 1.0 mmHg increase in DBP (95% CI: 0.3, 1.8) based on models adjusted for race/ethnicity and all model 3 covariates (data not shown). In fully adjusted models (model 3) that included interaction terms for race/ethnicity and log-transformed BPb, a doubling of BPb was associated with a 3.2 mmHg (95% CI: 1.5, 5.0) increase in SBP in blacks compared with a 1.0 mmHg (95% CI: –0.3, 2.4) increase in whites (β_interaction_ = 2.2 mmHg; 95% CI: 0.3, 4.1) (Table 2). However, associations between BPb and DBP were not significantly different between blacks and whites (1.8 mmHg; 95% CI: 0.7, 2.8 and 0.9 mmHg; 95% CI: 0.1, 1.8, respectively; β_interaction_ = 0.8 mmHg; 95% CI: –0.3, 2.0).

**Table 2:** Association [regression coefficient (95% CI)] between log-transformed BPb and BP (mmHg) by race/ethnicity, NHANES 2005–2008.

Model	Black (n = 1,218)	White (n = 3,252)	Black–white differencea
SBP
1b	3.2 (1.5, 4.9)	1.0 (–0.4, 2.3)	2.2 (0.3, 4.2)
2c	2.6 (0.8, 4.4)	0.6 (–0.8, 2.0)	2.0 (0.0, 4.0)
3d	3.2 (1.5, 5.0)	1.0 (–0.3, 2.4)	2.2 (0.3, 4.1)
DBP
1b	1.1 (0.0, 2.1)	0.7 (–0.3, 1.6)	0.4 (–0.7, 1.5)
2c	1.2 (0.1, 2.3)	0.8 (–0.2, 1.7)	0.4 (–0.7, 1.6)
3d	1.8 (0.7, 2.8)	0.9 (0.1, 1.8)	0.8 (–0.3, 2.0)
Results weighted to account for complex survey design. aRegression coefficient for the race/ethnicity × BPb (log-transformed) interaction term. bIn addition to race/ethnicity, BPb (log transformed) and race/ethnicity × BPb, model includes age, age2, and sex. cModel 1 plus education (< high school, high school, ≥ high school) and family PIR. dModel 2 plus hematocrit, BMI, heavy alcohol use, smoking status (never, former, current), and diabetes diagnosis.						

Models that included a three-way interaction suggested that associations between BPb and SBP were limited to blacks with high depressive symptoms, among whom a doubling of BPb was associated with a 5.6 mmHg (95% CI: 2.0, 9.2) increase in SBP, compared with a 1.8 mmHg (95% CI: 0.2, 3.5) increase among blacks with low depressive symptoms (β_interaction_ = –3.8 mmHg; 95% CI: –7.7, 0.2) (Table 3). Furthermore, at low levels of depressive symptoms, there was no significant racial/ethnic disparity in the association between BPb and SBP (β_interaction_ = 0.9 mmHg; 95% CI: –0.9, 2.7); whereas at high levels of depressive symptoms, there was a large and significant disparity (β_interaction_ = 4.4 mmHg; 95% CI: 0.5, 8.3).

**Table 3:** Association [regression coefficient (95% CI)] between BPb and BP (mmHg) by race/ethnicity and level of depressive symptoms, NHANES 2005–2008.

	Black	n	White	n	Black–white difference
SBP
Low depressive symptoms	1.8 (0.2, 3.5)	764	1.0 (–0.6, 2.6)	2,073	0.9 (–0.9, 2.7)
High depressive symptoms	5.6 (2.0, 9.2)	454	1.2 (–0.5, 2.9)	1,179	4.4 (0.5, 8.3)
Low DS–high DS difference	–3.8 (–7.7, 0.2)		–0.2 (–2.3, 1.8)
DBP
Low depressive symptoms	1.2 (–0.1, 2.4)	763	1.2 (0.2, 2.1)	2,073	0.0 (–1.1, 1.1)
High depressive symptoms	2.8 (0.9, 4.8)	454	0.4 (–0.7, 1.6)	1,179	2.4 (0.1, 4.7)
Low DS–high DS difference	–1.7 (–4.0, 0.6)		0.7 (–0.5, 2.0)
DS, depressive symptoms. Results weighted to account for complex survey design. In addition to lower order terms, pair-wise interactions and a three-way interaction term for race/ethnicity, depressive symptoms, log-transformed BPb, models include age, age2, education (< high school, high school, ≥ high school), family PIR, hematocrit, BMI, heavy alcohol use, smoking status (never, former, current), and diabetes diagnosis.										

A doubling of BPb among blacks with low and high depressive symptoms was associated with a 1.2 mmHg (95% CI: –0.1, 2.4) and 2.8 mmHg (95% CI: 0.9, 4.8) increase in DBP, respectively, (β_interaction_ = –1.7 mmHg; 95% CI: –4.0, 0.6) (Table 3). A doubling of BPb among whites with low and high depressive symptoms was associated with a 1.2 mmHg (95% CI: 0.2, 2.1) and 0.4 mmHg (95% CI: –0.7, 1.6) increase in DBP, respectively (β_interaction_ = 0.7 mmHg; 95% CI: –0.5, 2.0). Among those in the low depressive symptoms group, there was no racial/ethnic disparity in the BPb–DBP association (β_interaction_ = 0.0 mmHg; 95% CI: –1.1, 1.1). However, there was a significant disparity between blacks and whites in the high depressive symptoms group (β_interaction_ = 2.4 mmHg; 95% CI: 0.1, 4.7).

## Discussion

Our analysis shows that there continue to be racial/ethnic disparities in the association between BPb and BP based on recent NHANES data. Furthermore, the association between BPb and BP was strongest among blacks who reported high levels of depressive symptoms, consistent with the hypothesis that racial/ethnic disparities in psychosocial stress and social stressors result in an increased vulnerability to the health effects of environmental exposures (Gee and Payne-Sturges 2004). In other words, our findings suggest that social factors may be important determinants of environmental health disparities.

There is a growing literature suggesting that social stressors and psychosocial stress moderate the association between lead and health. For example, the association between traffic-related pollution and incident asthma was stronger for children whose parents reported high, compared to low, levels of perceived stress (Shankardass et al. 2009) and for children with high, compared to low, exposure to violence (Clougherty et al. 2007). Similarly, the association between traffic-related pollution and coronary artery calcification was reported to be stronger among adults living in neighborhoods with high, compared to low, unemployment (Dragano et al. 2009).

Although we did not test biological mechanisms, the notion that stress increases vulnerability to the hypertensive effect of lead is biologically plausible. Lead and psychosocial stress both affect biological pathways relevant to BP control, including the neuroendocrine and cardiovascular responses (Ahamed and Siddiqui 2007; Cory-Slechta et al. 2008; Harrison and Gongora 2009; Payton et al. 1993; Virgolini et al. 2005, 2006, 2008). Animals and their offspring had higher glucocorticoid levels when exposed to both restraint stress and lead than animals exposed to either restraint stress or lead alone (Cory-Slechta et al. 2004; Virgolini et al. 2006). These results are consistent with a synergistic effect of lead and stress on biologic responses to stress.

The exact mechanisms of how lead and stress potentiate each other’s health effects are not known. However, one possible mechanism is that either chronic stress or lead—or both—may chronically activate the biological stress response systems. This chronic activation may increase wear and tear—or allostatic load—on the neuroendocrine and related cardiovascular systems. This allostatic load state is characterized by a lack of appropriate control of the cardiovascular system, resulting in hypertension (McEwen and Seeman 1999; Seeman et al. 1997). Normally, cortisol increases in response to a stressor and then decreases with the removal of the stressor, and children with low BPb levels (1 SD below the mean) show this pattern of cortisol increase and decrease in response to a laboratory mirror-tracing stressor in which participants used a computer mouse and cursor to trace a star on the computer screen (Gump et al. 2008). However, children with high BPb levels (1 SD above the mean) did not exhibit a cortisol decrease after cessation of the stressor task (Gump et al. 2008). In our study, depressive symptoms, measured by the PHQ-9, may mark the allostatic load of the stress response system (McEwen 2003), which then results in a heightened hypertensive response to recent low lead exposure, as marked by lead measured in blood. Together, this suggests that both psychosocial stress and lead wear overburden the stress response systems making further proper response to stressors and environmental hazards difficult.

Our study had some limitations, including the use of BPb as the marker of lead exposure instead of bone lead, which provides a better indication of lifetime lead exposure and may better capture lead’s effect on health than BPb (Hu 1998). Because NHANES contains only BPb, we limited our BP outcomes to SBPs and DBPs, which can change in response to short-term exposures. Future research using bone lead as a measure of exposure should consider outcomes that reflect more chronic pathophysiological processes, including pulse pressure, a marker of arterial stiffness, and hypertension.

Another limitation was the use of depressive symptoms as a proxy marker for psychosocial stress. Although there is evidence to suggest that depressive symptoms are a consequence of chronic psychosocial stress, they are only an indirect and crude proxy for stress (Aneshensel et al. 1991; Mirowsky and Ross 2003). Further research would benefit from the use of more direct stress measures. Perceived chronic stress, in particular, may explain the racial/ethnic disparity in the association between BPb and BP, as perceived stress captures the extent to which stressors are actually stressful and thus engaging the biological stress processes. Finally, NHANES is a cross-sectional study and we were not able to examine temporal relations between BPb, depressive symptoms, and BP changes.

More research is needed that incorporates both social and environmental factors when examining racial/ethnic health disparities. Furthermore, a growing body of literature suggests that health disparities are produced and maintained through the cumulative effects of social and environmental factors (Espinosa et al. 2004; Morello-Frosch and Lopez 2006; Morello-Frosch et al. 2011). Although we were able to empirically evaluate one socially related factor (depressive symptoms) and one environmental hazard (BPb), health disparities are more likely the result of multiple social factors (e.g., psychosocial stress, poverty, discrimination) and multiple environmental exposures (e.g., lead, air pollution, noise pollution). More research is needed that incorporates multiple social and environmental exposures.

## Conclusions

Our research contributes to a growing literature addressing the notion of differential vulnerability as it pertains to environmental health disparities, and is one of the first empirical examinations of differential vulnerability in the association between BPb and racial/ethnic disparities in hypertension. With our research, we respond to the call to integrate social factors into research on environmental health disparities (Gee and Payne-Sturges 2004; Hicken et al. 2011). Our findings reinforce the value of moving beyond traditional notions of environmental health by integrating social and psychological factors into research on the role of environmental hazards in health disparities.
